# A Call to Future-Proof Environmental Science and Engineering

**DOI:** 10.1021/acs.est.6c11422

**Published:** 2026-10-13

**Authors:** Kristopher McNeill, Alexandria B. Boehm, David M. Cwiertny, Heileen Hsu-Kim

**Affiliations:** † Institute of Biogeochemistry and Pollutant Dynamics, ETH Zurich, 8092 Zurich, Switzerland; ‡ Department of Civil and Environmental Engineering and Department of Oceans, 6429 Stanford University, Stanford, California 94305, United States; § IIHR Hydroscience and Engineering, 4083 University of Iowa, 300 South Riverside Drive, Iowa City, Iowa 52246, United States; ∥ Department of Civil and Environmental Engineering, University of Iowa, Iowa City, Iowa 52246, United States; ⊥ Department of Chemistry, University of Iowa, Iowa City, Iowa 52246, United States; # Center for Health Effects of Environmental Contamination, University of Iowa, Iowa City, Iowa 52246, United States; 7 Department of Civil & Environmental Engineering, 3065 Duke University, Durham, North Carolina 27708, United States

**Keywords:** future-proofing, policy, practice

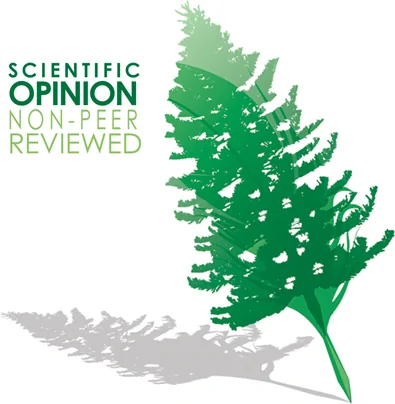

Socializing at a conference, a group of us were talking. One of us said to the others:

“The field of environmental science and engineering may be dying. We have solved all the big problems. We know how to remove nutrients and pathogens from wastewater and treat drinking water. We’ve been doing this for decades. Now all we do is chase research funding. And now that is totally insecure, especially in the U.S.”

This is a cynical perspective of our field and its future, but nonetheless, one that contains some serious criticism worthy of reflection. A more affirmative view is that environmental science and engineering researchers are nimble and responsive, able to move easily from one problem to the next. Environmental science and engineering scholars have been heavily involved in responses to nearly every environmental crisis over the past several decades, from those caused by natural disasters (e.g., Hurricane Katrina, 2004 Indian Ocean tsunami, and 2010 Haiti earthquake) to those caused by humans (e.g., Deepwater Horizon oil spill) and harmful contaminants in water (e.g., arsenic, lead, and PFAS). We collaborate with communities and public agencies around the globe to solve real environmental problems; these activities are rewarding and yield tangible benefits to society.

However, our willingness to pivot quickly to address immediate problems can cause our research focus as a discipline to shift wildly. Ever-changing priorities divert funding resources from old to new problems, luring us into different research directions and leaving unresolved voids in legacy focal areas. This approach also instills a perception, particularly in early career scholars, that our community is guided simply by the pursuit of research dollars thrown at the latest shiny problem. We readily admit that our own research portfolios have followed such paths. One of us shifted their own research program to focus on pesticides, PFAS, and microplastics. Another shifted their work to wastewater-based epidemiology in response to research needs and funding availability during the pandemic. Yet another shifted research from toxic metals to nanomaterials to critical minerals resourcing.

At certain moments, funding resources and emerging environmental problems are shaping research and education agendas in our field, and our reputations as scholars, in what feels to be an *ad hoc* manner. Is this a problem? We think so. As the allocations of limited extramural and institutional resources continue to focus on emerging areas, new discoveries that reinforce the discipline and offer foundational scientific advancement are at risk of stagnation or forgotten in educational programming. This mode of scholarly pursuit for the discipline is especially vulnerable to research investments that fluctuate according to economic and political circumstances.

Now seems like an important time to take stock in what is important to our field and try to future-proof our research and education programs.

We engaged 55 researchers, teachers, and practitioners for an in-person workshop discussion of these issues at the 2025 Research and Education Conference for the Association of Environmental Engineering and Science Professors (AEESP), held in May 2025 in Durham, North Carolina. While the membership of AEESP represents only a subset of our research community, the workshop provided an outstanding opportunity to engage a large portion of the U.S.-based research community in a conversation that we hope will continue with our colleagues around the globe. We sought to discuss the key anchors of our education and research programs, as well as pathways to positively influence funding priorities and strategies that improve public perceptions of our field. Half of the workshop participants were university faculty, while the other half were research trainees (student and postdoctoral) or practicing environmental engineers. The participants were asked to identify, in small group discussions, the pillars and cross-cutting foci for research and education programs, recommendations for influencing policy, funding and engineering practice, and approaches to promote and communicate the value of our discipline to an audience outside academia.

The group was unanimous that future-proofing our research and education programs requires sustained support for disciplinary fundamentals, even as we provide interdisciplinary training in emerging cross-cutting areas. Fundamentals include environmental chemistry and microbiology, fluid mechanics and hydrology, and unit process design. Engagement of the current generation of students should include classes that teach these core concepts, underpinned by principles of energy and mass balances, toward salient real-world applications such as water and wastewater treatment, air pollution control technologies, biogeochemical cycling, and climate science. The group identified cross-cutting areas of systems science and engineering, data science including contemporary AI tools, social science, and public health, epidemiology, and toxicology. A unique aspect of environmental science and engineering is the strong connection to real-world environmental challenges in the private and public sector, so there is a need to prepare students with practical problem-solving and communication skills. There is also a need to develop an appreciation for the ethical and political contexts of environmental science and engineering.

The group made clear that influencing policy, funding, and engineering practice are essential translational activities in our field that set it apart from the natural sciences. It is through these activities that our work has broad, positive societal benefits. To do so, we need to distill scientific findings into nontechnical white papers and policy briefs that can be understood by elected officials and their staff, leadership at local, state, and federal agencies, and engineering practitioners. The importance of cultivating relationships with individuals with decision-making authority cannot be overstated. Industry and private sector partnerships facilitate the transfer of new science from academia while teaching academics about entrepreneurship. Student interns embedded in agencies and industries foster collaborative exchange of ideas. Professional education opportunities can bridge the academic–practice divide. For example, the U.S. National Science Foundation offers research grant programs (e.g., NSF INTERN and I-PhD) to directly support student experiences in public agencies or in the private sector while obtaining a Ph.D.

The workshop participants also acknowledged that the public is not well informed about the fields of environmental science and engineering. Communication efforts aimed at the public, of all ages, can address this. Research groups with media engagement opportunities can promote not only their own research findings but also our field (e.g., by explicitly mentioning “environmental engineering” during interviews). Strong communicators should engage with the public through social media as environmental science and engineering influencers, including through centrally coordinated efforts. Creative communication approaches could involve storytelling through comic books, board games, music, or art. Engagement of children in communication efforts was identified as important.

Advocacy and engaged science were identified as critical responsibilities of members of our community. Here, advocacy refers to efforts to promote scientific findings to outside audiences while maintaining academic impartiality. Engaged science refers to science in communities that involves a genuine, two-way dialogue between researchers and people impacted by the research; it involves listening and speaking.

Some examples of specific action items offered by workshop attendees are summarized in Table 1. These have been organized based upon recommendations derived from the workshop and summarized above: sustaining support for disciplinary fundamentals; approaches to influence policy, funding, and engineering practice; communication efforts aimed at the public; and actions to support advocacy and engaged science within our discipline. Although far from comprehensive, we hope the ideas in Table 1 can be a starting point for building a stronger, more future-proof discipline.

**Table 1:** Strategies and Example Actions Identified during the 2025 AEESP Workshop “Charting the Future” Held at Duke University, Durham, North Carolina, USA

strategic area	example actions
sustain disciplinary fundamentals	• advocate for departmental hiring that maintains disciplinary health
	• include fundamentals explicitly in course delivery
	• encourage students to take courses that provide disciplinary foundations
influence policy, funding, and engineering practice	• engage with government officials and congressional staff
	• develop and disseminate policy memos and white papers
	• build relationships with key partners and stakeholders outside academia
	• present at industry and trade conferences
	• seize unusual opportunities for engagement
communicate with the public	• create a podcast
	• develop and maintain a social-media presence
	• establish relationships with local reporters and social-media influencers
	• write op-eds
	• use unconventional formats, such as comics, to communicate research
support advocacy and engaged science	• leverage unconventional partnerships
	• create reward structures (e.g., tenure requirements) for advocacy, engaged research, and translational science
	• build trust through interactive, two-way conversations
	• participate in citizen science
	• find your voice and share ideas broadly

We need a multipronged approach to prepare our students and our field for the future. We need to maintain fundamentals and train in new, emerging cross-cutting areas, and we need to preserve our close connection with those beyond academia and continue to address real-world problems. A coordinated approach for outreach, translation, and communication is also essential to ensure that decision makers and the public can appreciate the contributions environmental engineers and scientists make and the benefits our work brings to society. We hope that research and education programs across the country will embrace and implement these ideas so that our field can continue to contribute beneficial outcomes and services for society.

No, environmental science and engineering is not a dying discipline, but it is a discipline at risk of being pulled too easily from one urgent problem to the next without sufficient attention to the foundations that make those responses possible. The fact that our field can pivot rapidly is one of its strengths. The danger is that, without a clear articulation of our core principles, public value, and long-term intellectual agenda, that strength can look from the outside—and sometimes from the inside—like aimlessness.

The way forward is neither to resist emerging problems nor to retreat into a narrow definition of the field. Rather, we need to hold both commitments at once: strong disciplinary foundations in environmental chemistry, microbiology, hydrology, fluid mechanics, and engineering design, together with the capacity to engage new challenges in public health, climate change and energy systems, resource recovery, and environmental justice.

The provocative claim that opened this Viewpoint contains a warning, but not a diagnosis. Environmental science and engineering is not dying because the big problems have been solved. It is needed precisely because environmental problems keep emerging, combining, and reappearing in new forms. If we want the field to remain coherent, visible, and essential, we must do more than follow the problem of the moment. We must sustain the core subdisciplines that make environmental science and engineering capable of meeting moments we cannot yet predict.

